# Anticipating Disaster: Empowering Caregivers as Essential First Responders

**DOI:** 10.1093/geroni/igaf122.3540

**Published:** 2025-12-31

**Authors:** Díana Poncé, Jarmin Yeh, Kattia Suarez Vargas, Melinda Neri

**Affiliations:** University of California, San Francisco, San Francisco, California, United States; University of California, San Francisco, San Francisco, California, United States; University of California, San Francisco, San Francisco, California, United States; University of California, San Francisco, San Francisco, California, United States

## Abstract

Older adults and adults with disabilities face increased risks during climate disasters, like wildfires, and depend heavily on caregivers for physical and health-related support. Emergency evacuations during these events are particularly challenging due to mobility limitations and cognitive impairments, making caregivers critical in ensuring their care recipients’ safety. Yet, many caregivers may be underprepared to respond effectively without prior training. To address this gap, we conducted semi-structured in-depth interviews with 25 Medicaid-funded In-Home Supportive Services caregivers in San Bernardino County, California, who participated in an Emergency and Disaster Readiness Training between June 2024 and March 2025. The interviews explored caregivers’ experiences with mitigating risks and preparing for disasters, focusing on how they applied the training skills and concepts in practical scenarios. Through thematic analysis, our findings revealed that caregivers played a proactive role in safeguarding their care recipients by anticipating disaster risks and implementing preparedness measures. Notably, caregivers recognized their role as experts about their care recipients’ needs and as essential first responders in preparation for the arrival of official emergency services. They employed three key approaches to mitigate risks: modifying their care recipients’ homes to reduce the potential for injury, preparing and rehearsing emergency evacuation plans, and personalizing and supplementing emergency kits to address their care recipients’ specific needs. These findings highlight the importance of specialized training programs and policies to empower caregivers as essential first responders. By equipping caregivers with skills and resources to protect vulnerable populations, we can enhance disaster preparedness and improve outcomes for older adults.

